# NINE-YEAR ASSOCIATION OF OPTIMISM AND COGNITIVE FUNCTIONING IN A COHORT OF AGING MEN

**DOI:** 10.1093/geroni/igad104.0943

**Published:** 2023-12-21

**Authors:** Victoria Marino, Samsuk Kim, Avron Spiro, Lewina Lee

**Affiliations:** Boston University Chobanian & Avedisian School of Medicine, Boston, Massachusetts, United States; The VA Boston Healthcare System, Boston, Massachusetts, United States; VA Boston Healthcare System, Boston, Massachusetts, United States; Boston University School of Medicine, Boston, Massachusetts, United States

## Abstract

Higher levels of optimism have been linked to better physical and emotional health, and growing evidence suggests optimism may also protect against cognitive impairment and decline. However, our understanding of its prospective associations with cognitive functioning is limited. We examined 9-year associations of optimism with cognitive functioning among 793 men from the VA Normative Aging Study. Optimism was assessed via the Minnesota Multiphasic Personality Inventory-2 in 1986 (baseline: AgeM = 60.1, SD=7.1). Cognitive functioning was measured at participants’ earliest cognitive testing occasion between 1993 and 1996. We considered both global cognition (MMSE) and three domains of cognitive functioning: executive function (verbal fluency via Animal Naming Test, working memory via WAIS-R Digit Span Backward), short-term memory (immediate and delayed CERAD Word List Recall), and visuospatial ability (CERAD and VMI spatial copying, NES-2 Pattern Comparison). Analyses used ordinary least squares regression adjusted for childhood socioeconomic status, and demographics and major diseases at baseline. Optimism was not associated with global cognition. However, higher optimism was associated with better verbal fluency (B=0.48, SE=0.19, 95% CI .11–.86, p=.01) and immediate word list recall (B=0.33, SE=0.15, 95% CI .04–.62, p=.02), and weakly associated with delayed word list recall (B=0.14, SE=0.07, 95% CI -.01–.28, p=.06). Optimism was not associated with visuospatial ability or working memory performance. Our findings suggest that optimism could be particularly important for verbally mediated aspects of executive functioning and memory in aging men. Optimism may be a valuable intervention target to promote healthy cognitive aging.

